# Lock-In Thermography with Cooling for the Inspection of Composite Materials

**DOI:** 10.3390/ma16216924

**Published:** 2023-10-28

**Authors:** Ryszard Dymitr Łukaszuk, Rafael Monteiro Marques, Tomasz Chady

**Affiliations:** 1 Doctoral School, West Pomeranian University of Technology, 70-313 Szczecin, Poland; ryszard.lukaszuk@zut.edu.pl; 2 Independent Researcher, Faculty of Electrical Engineering, West Pomeranian University of Technology, 70-313 Szczecin, Poland; rafael.monteiro2ms@gmail.com; 3 Faculty of Electrical Engineering, West Pomeranian University of Technology, 70-313 Szczecin, Poland

**Keywords:** nondestructive testing (NDT), nondestructive evaluation (NDE), lock-in thermography (LIT), glass fiber-reinforced composites

## Abstract

This paper presents the development of the lock-in thermography system with an additional cooling system. System feasibility is tested by investigating a square-shaped glass fiber-reinforced polymer (GFRP) with artificially made outer flaws. The influence of heating mode and sinusoidal excitation period on the defect detectability is considered. Thus, the experiment is split into two modes: the sample is solely heated in the first mode or simultaneously heated and cooled in the second. In each mode, the temperature measurement is performed first with a shorter excitation signal period and second with a longer one. The signal-to-noise ratio (SNR) is used to assess defect detection quantitatively. The comparative analysis shows that employing a mixed heating–cooling mode improves the SNR compared to the conventional heating mode. The further enhancement of the SNR is obtained by extending the excitation period. The combination of simultaneous heating and cooling with longer periods of the excitation signal allows for the best SNR values for the most detected defects.

## 1. Introduction

A composite is a hybrid material assembled from two or more materials with different physical and chemical properties. Composites are gaining prominence over other traditional materials due to their excellent high strength-to-thickness ratio, cost-efficient manufacturing, wear resistance, low thermal expansion, and ease of customization and assembly [1,2]. However, composites exhibit high anisotropy and inhomogeneity. The continuous improvement and development of composite manufacturing methods and unique material properties has led to the widespread application of composites in modern industry branches, such as the automotive (body material, bumpers, fuel tanks) [3,4,5], marine (decks, hulls, propellers) [6,7,8], aviation (fuselages, stabilizers) [9,10,11], offshore (pipelines, structure reinforcements) [12,13,14], power engineering (wind turbine poles and blades) [15,16], and civil engineering (hydraulic structures, building claddings, maintenance holes, reinforcements) industries [2,17]. Moreover, composites are successfully used in biomedicine (dental and surgical implants, blood vessels, bone fillers) [17] and sports (cycling and sailing equipment).

During their lifetime, composites are subject to various impacts that may compromise their structural integrity [18]. Particularly hazardous are subsurface changes invisible to the human eye [19]. Independent from the composite type, manufacturing-induced inhomogeneities may occur between layers (delamination, un-infiltration), in the resin matrix (resin-rich area, void, impurity, porosity), and the fiber reinforcement (fiber misalignments such as waviness or wrinkling, breakage). Delamination is considered one of the most dangerous composite defects because it develops without any externally visible factor. Besides the improper conditions of curing during the manufacturing process of the composite, pressure or stress load acting on the component during its service use may also instigate delamination. Another type of defect is a void. This may appear due to air intrusion during the molding process. The exact process may also contribute to the formation of resin-rich zones, which result from poor fiber integration and act as the residual stress source. Misalignment encapsulates defects such as waviness, wrinkling, brokenness, undulation, and folding. Fiber misalignments appear as a result of vulnerable impacts during various manufacturing processes. All these flaws threaten a composite’s properties and performance [20,21,22].

Therefore, it is vital to examine the condition of composite materials by detecting and identifying inhomogeneities at the earliest stages of their formation to minimize the risk of component catastrophic failures, massive economic loss, and personnel injuries. Nondestructive testing (NDT) methods respond to the demand for safety and quality assurance and the long-term exploitation of composite structures. Several modalities are successfully used to inspect composite materials, such as terahertz spectroscopy, ultrasonic method, shearography, X-ray, and thermography, to name a few.

The terahertz method (THz) is suitable for localizing surface and internal inhomogeneities, such as intrusions, moisture, voids, or delamination. The inspection procedure does not require a coupling medium and is safe for personnel because of nonionizing radiation [23]. However, this method is restricted to nonconductive materials because of high terahertz wave attenuation in conductors [24].

The ultrasonic method emits acoustic waves into the examined structure using a transmitter. This technique detects delamination, matrix breakages, and wrinkles [25,26]. The traditional methodology presents issues, such as the need to apply a coupling agent [27]. For this reason, air-coupled and laser ultrasonic methods are under development. The undeniable merits of these approaches include a noncontact procedure and no need to use coupling [28].

Shearography is a digital optical interferometric method that detects abnormalities like impact damage, fiber cracks, and delamination [29,30]. Shearography is profitable due to the time-efficient, full-field, and noncontact procedure. One drawback is that the stress level to be induced in the examined object has to be chosen accurately and carefully to avoid structural damage [31].

In X-ray testing, ionizing radiation is used to transmit X-rays through the examined structure. This technique allows the detection of abnormalities such as porosities, inclusions, or voids. The advantages of X-rays comprise a noncontact measuring procedure, high measurement resolution, and sensitivity. A significant disadvantage is the hazardous ionizing radiation [32].

Active infrared thermography (IRT) is an advantageous and promising approach for evaluating the condition of composite materials [33]. It offers a full-field, safe-to-perform, reliable, noncontact, accurate, cost-effective, and portable inspection procedure [34,35]. During the inspection, an excitation source emits heat absorbed by the surface of the structure under inspection. Heat waves propagate inside the material, partially dissipate, and reflect from encountered inhomogeneities. The resultant reflected waves interfere with the waves originating from the excitation source [19,36]. Consequently, an infrared camera can observe and register surface temperature variations correlating with internal abnormalities. Various external excitation sources, such as light, mechanics, and microwaves, can be used for heat induction in the tested material.

Optical thermography utilizes flash lamps or lasers as an external heat excitation source. Regarding excitation mode, optical thermography is classified as lock-in (LIT) and pulse (PT). In lock-in thermography, the heating signal emitted to the structure is periodic and amplitude-modulated, usually a square or sine wave. It is advantageous because the gradual heating process reduces the hazard of thermal-induced damage, and the signal-to-noise ratio is relatively high [37]. One drawback is that the resultant thermograms are vulnerable to numerous distortions that must be diminished during signal postprocessing [38]. On the contrary, pulse thermography employs a signal composed of short rectangular pulses. This excitation mode excessively depends on non-uniform heating and surface emissivity [39].

Vibrothermography (VT) uses ultrasonic waves to induce heating in the area of interest. When an ultrasonic wave approaches an inhomogeneity, mechanical energy is transferred into a heat wave, then captured by an infrared camera [40]. This technique has several advantages, such as ease of measuring procedure or adaptability to various component shapes [41].

Induction thermography (IT) can be applied to metallic materials and carbon fiber-reinforced polymers. In this technique, an induction coil produces a magnetic field penetrating the examined structure and induces eddy currents. Abnormalities in the inspected component disturb the eddy current paths. As a result, heat is generated and observed by an infrared camera [42].

In microwave thermography, microwaves act as a heating excitation source. If a structure to be tested is dielectric, the amount of dissipated heat depends on the permittivity of the material, microwave frequency, and the electric field magnitude. This approach is beneficial because the energy delivered to the examined component is of medium power and thus does not cause sudden temperature rises, which are potentially harmful to the structure [43].

After thermography inspection, it is imperative to handle image postprocessing to reduce the impact of the background noise and infrared camera influence on the results and thus improve the detectability of the material inhomogeneities and extract amplitude and phase images [1,44]. To achieve this, numerous algorithms are employed. Conventionally, noise reduction may be performed using spatial or frequency-domain filtering. Spatial filtering involves, e.g., arithmetic mean or median filters [45]. Frequency-domain filtering relies on applying low-pass, high-pass, or band-pass filters.

There has been intensive research in lock-in thermography for modern material evaluation. Vesala et al. proposed a lock-in thermography system with a deep anomaly detection model and successfully tested it on CFRP and GFRP structures with artificial defects. The authors used a CFRP sample with 25 flat-bottom hole defects of different diameters, namely 16 mm, 14 mm, 10 mm, 8 mm, and 4 mm, and depths, namely 0.2 mm, 0.5 mm, 0.8 mm, 1.1 mm, and 1.5 mm. A 0.01 Hz–0.1 Hz frequency sweep modulated the heat excitation flux [46]. Dong et al. performed a nondestructive inspection of samples made from CFRP, steel, and aluminum alloy using a reflective lock-in thermography system. The training sample contained 24 flat bottom hole defects with 2 mm, 5 mm, 10 mm, and 15 mm diameters. The defect depths were 0.5 mm, 0.8 mm, 1 mm, 1.2 mm, and 1.5 mm. As an excitation source, two 1000 W halogen lamps were utilized. The excitation flux was modulated with the frequencies 0.025, 0.05, 0.075, and 0.1 Hz [44]. Sapieta et al. focused on detecting flat-bottom holes in additive-manufactured samples made of PET-G. The defects’ depths were 0.5 mm and 1 mm. The circular defects had a diameter of 20 mm, while the square-shaped had an edge length of 20 mm. The researchers used two types of excitation, flash lamps and halogen lamps, with heat excitation periods of 60 s and 120 s, respectively [19]. Cheng et al. developed a system for automatically detecting rectangular flat-bottom holes in CFRP. They examined a sample with 16 defects of different depths, namely 1.6 mm, 1.9 mm, 2.3 mm, and 2.6 mm, and edge lengths, namely 7 mm, 10 mm, 13 mm, and 16 mm. The authors employed a system composed of two 1000 W lamps with the excitation flux modulated with a frequency of 0.05 Hz [1].

This study focuses on the influence of different heating/cooling modes and excitation periods on the detectability of relatively small-diameter flat-bottom holes embedded in a GFRP sample.

## 2. Materials and Methods

Of the various techniques originating from thermography, lock-in thermography (LIT) has garnered considerable attention because of the possibility of detecting structural abnormalities quantitatively [47]. During the inspection, an external sinusoidally or square-wave-modulated light source, such as halogen lamps, emits heat absorbed by the structure’s surface under examination. Heat waves propagate inside the material, partially dissipate, and reflect off encountered inhomogeneities. The resulting reflected waves interfere with those originating from the excitation source [19,36]. Consequently, an infrared camera can observe and register surface temperature variations. Several signal processing techniques for extracting thermal wave parameters are suitable: the four-point correlation method (FPCM), digital lock-in correlation method (DLCM), and fast Fourier transform (FFT). In the FPCM, four data points of the surface temperature, with the same time intervals between each other, have to be selected. The outcome comprises amplitude and phase images. The DLCM employs the correlation between a measured sinusoidal thermal wave and two reference sine/cosine functions to retrieve the amplitude and phase of the measured signal [48]. Fast Fourier transform extracts signal parameters by discretizing a thermogram, analyzing amplitude/phase in the frequency domain, and utilizing inverse Fourier transform [49].

LIT requires considering several parameters to obtain an acceptable signal-to-noise ratio value (SNR), such as excitation frequency; the number of heating periods; and the distances between the camera, the heating source, and the object to be tested [50].

**Figure 1:**
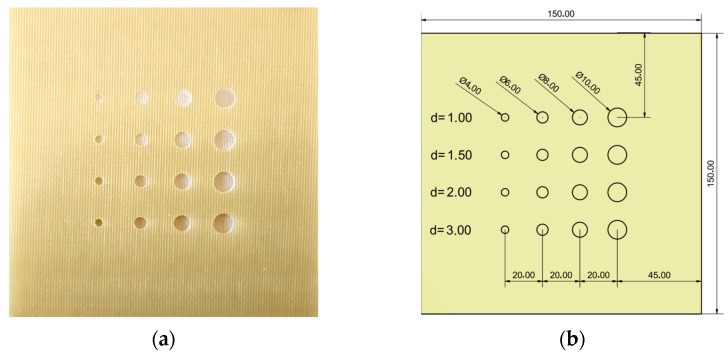
A square-shaped GFRP sample with flat-bottomed holes: (**a**) photo of the sample; (**b**) scheme of the sample.

This study uses a square-shaped GFRP (glass fiber-reinforced plastic) sample (150 mm × 150 mm × 4 mm) with flat-bottomed holes (Figure 1a). The flat-bottom holes have 10, 8, 6, and 4 mm diameters and depths of 1, 1.5, 2, and 3 mm (Figure 1b).

**Figure 2:**
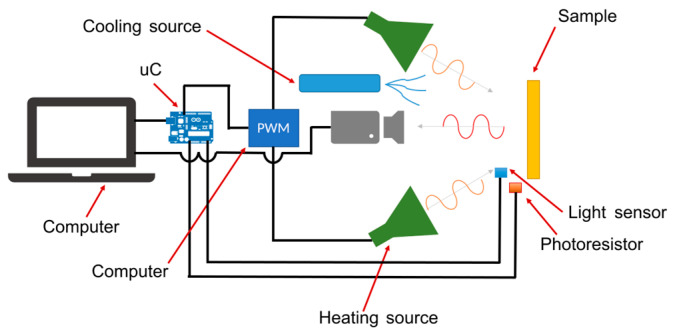
A schematic view of the measuring system.

**Figure 3:**
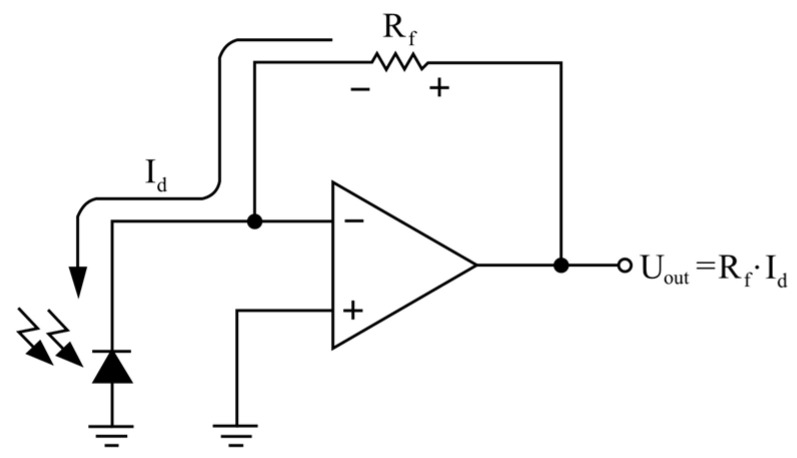
Circuit of the light (IR) sensor.

This study employs a self-made reflective LIT system consisting of a computer, a microcontroller (uC) Arduino Micro, a PWM unit, a cooling unit, two halogen lamps, an infrared camera, and a photoresistor with an electronic circuit. The computer programs the uC to drive the PWM unit. The PWM unit is supplied with two 300 W halogen lamps. The lamps produce sinusoidally modulated thermal waves for the heat excitation of the material to be tested. The TE-EQ1 uncooled infrared camera was placed in the front of the sample and used to register a thermal response from the sample’s surface. The camera faces the unflawed side of the samples so that the visible defects are hidden from the camera. An infrared sensor composed of an IR photodiode BP104 and an operational amplifier TDA2822M was used to establish an optimal heating source location. The irradiation from two halogens measured in front of the sample was over 60 W/m^2^. A schematic view of the measuring system is depicted in Figure 2, while the light (IR) sensor’s circuit is shown in Figure 3. The camera parameters are presented in Table 1.

## 3. Measurement

**Figure 4:**
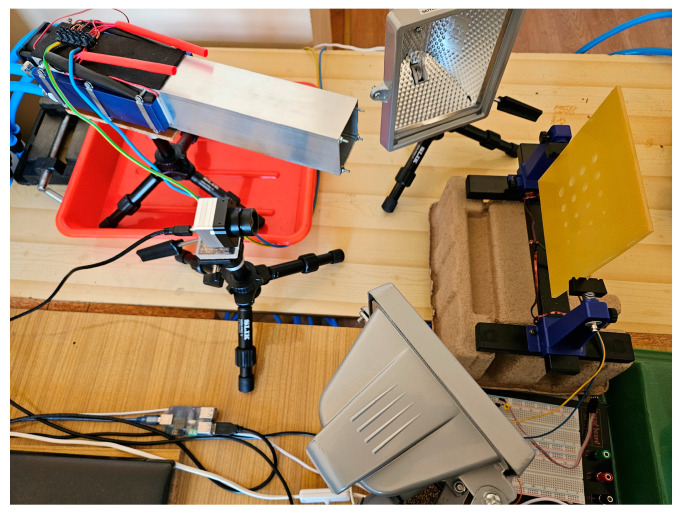
A photo of the measuring system.

**Figure 5:**
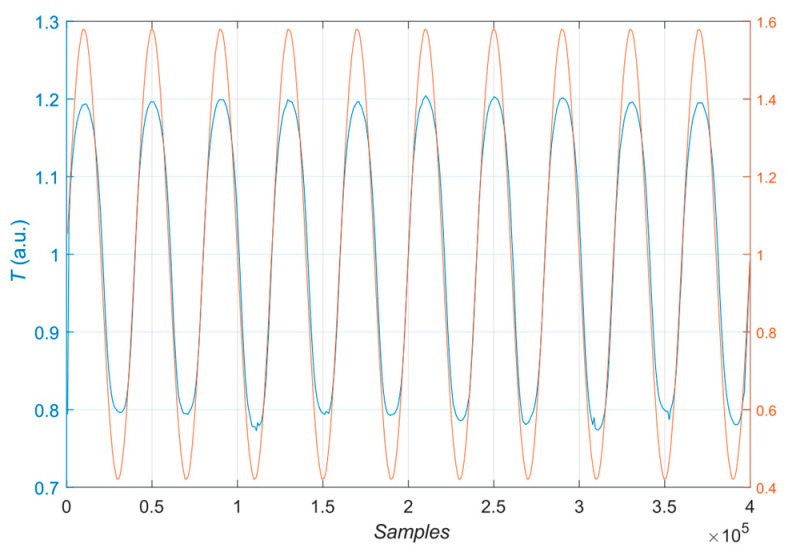
Normalized signals: controlling the PWM unit (orange curve) and measured using the photoresistor (blue curve).

The study used two modes: heating mode with halogen lamps and mixed mode: parallel heating with halogens and chilling with a cooling unit. During the selected experiments, the custom-made cooling unit was utilized to lower the temperature of the sample by continuously blowing air cooled by four Peltier elements. The Peltier elements are cooled by running water to enhance cooling efficiency. The average temperature of the cooled air was around −1 °C. On the one side, the Peltier element has a lower temperature than the environment; on the other, the heat is dissipated. The component allows a reduction in temperature of about 20 °C, and regarding the temperature of the water cooler, the reduction is about 15 °C. A photo of the measuring system is presented in Figure 4. An example of a signal controlling the PWM circuit and a signal corresponding to the radiation of thermal intensity measured by the photoresistor is depicted in Figure 5.

**Figure 6:**
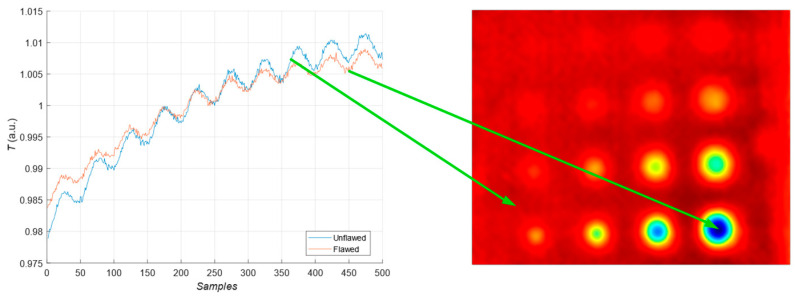
Exemplary normalized thermal response for the flawed (orange curve) and unflawed (blue curve) region of the sample and a thermogram.

During the heating procedure, the average temperature increases on the entire surface. Figure 6 presents two exemplary thermal response signals: one for the unflawed region of the sample under test and one for the flawed. It can be concluded that the temperature rise in the case of flaws is slower than for the healthy parts of the sample.

## 4. Results

The results of the measurements are presented as follows: Section 4.1 contains the results obtained for an excitation period of *T* = 40 s, and Section 4.2 contains those for an excitation period of *T* = 100 s. For each period, the two versions were presented: the left column contains the results for the sample tested in the heating mode and the right column contains the results for the sample tested using the parallel heating–cooling mode. For both test modes, the following graphs are presented: heating curves of the damaged and undamaged areas of the specimen, the real and imaginary parts of the thermal response, the average amplitude and phase of the thermal response, and the peak-to-peak amplitude of the sinusoidal waveforms acquired for each pixel.

### 4.1. Experiment with an Excitation Period of T = 40 s

**Figure 7:**
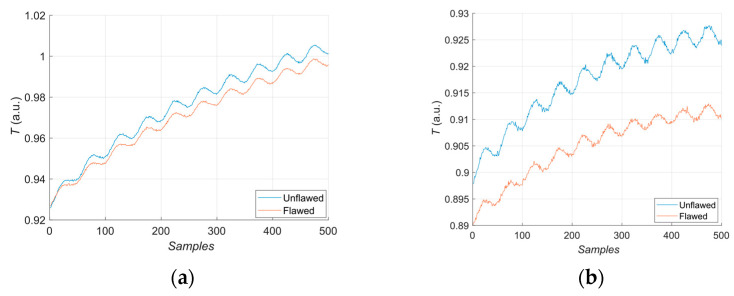
Thermal response for the flawed: (**a**) uncooled sample; (**b**) cooled sample. Excitation Period of *T* = 40 s.

**Figure 8:**
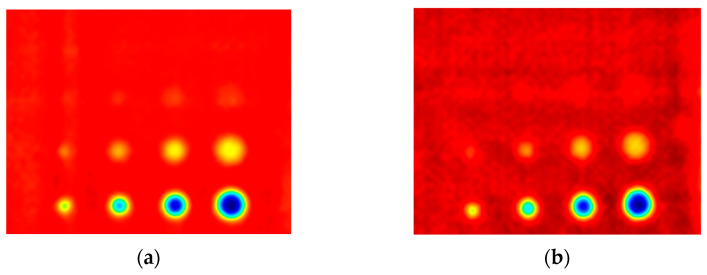
The real part of the signal: (**a**) uncooled sample; (**b**) cooled sample. Excitation Period of *T* = 40 s.

**Figure 9:**
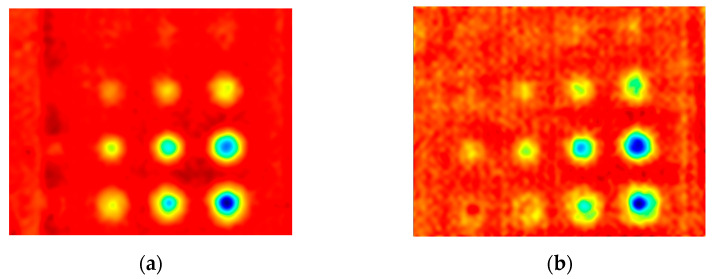
The imaginary part of the signal: (**a**) uncooled sample; (**b**) cooled sample. Excitation Period of *T* = 40 s.

**Figure 10:**
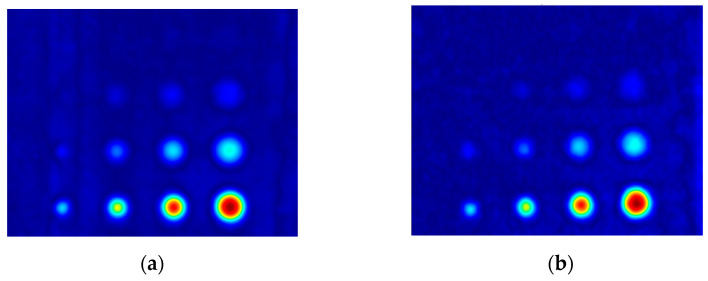
The mean amplitude of the signal: (**a**) uncooled sample; (**b**) cooled sample. Excitation Period of *T* = 40 s.

**Figure 11:**
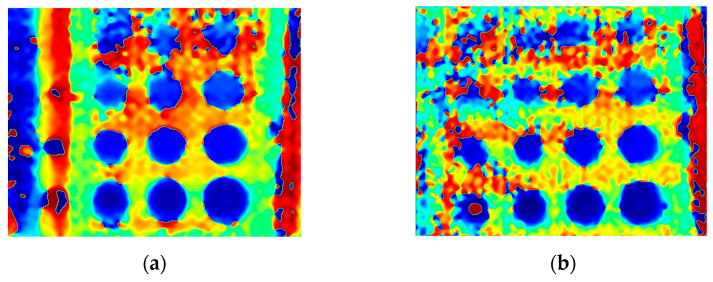
The mean phase of the signal: (**a**) uncooled sample; (**b**) cooled sample. Excitation Period of *T* = 40 s.

**Figure 12:**
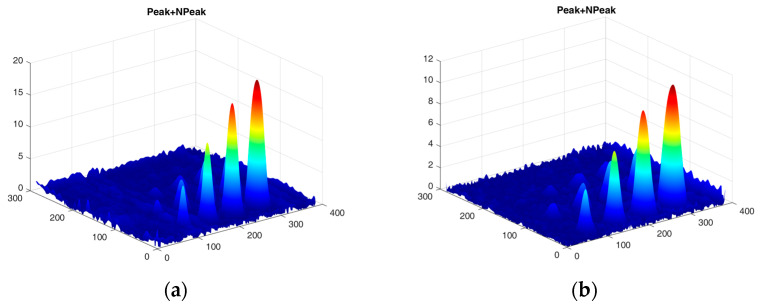
The peak-to-peak amplitude: (**a**) uncooled sample; (**b**) cooled sample. Excitation Period of *T* = 40 s. The colors correspond to the signal’s amplitude value.

The first experiment chose an excitation period of *T* = 40 s. Figure 6 depicts exemplary thermal responses for the flawed and unflawed regions of the sample under testing conditions. In the case of an uncooled sample, the average temperature rises very quickly in both the damaged and undamaged parts (Figure 7a). In contrast, the temperature rise for the simultaneously heated and cooled sample is slower, and the temperature values are much smaller, which is particularly evident for the curve corresponding to the damaged part of the sample (Figure 7b). Figure 8 contains graphs of the real part of the lock-in signal. As shown in Figure 8a, only defects with the largest diameter are detectable. The simultaneous heating and cooling process improves the detectability, as evident in Figure 8b. The following Figure 9 illustrates the imaginary part of the lock-in signal. The image for the sample without cooling (Figure 9a) highlights only 9 of the total 16 flaws. Cooling improves the defects’ detectability (Figure 9b); thirteen flaws are evident. Figure 9 comprises the average amplitude of the lock-in signal. It does not show significant differences in defect detection between the mode without cooling (Figure 10a) and the mode with cooling (Figure 10b). Figure 11 includes the phase shift of the lock-in signal. The image for the uncooled sample (Figure 11a) is partially indistinct and does not allow for the identification of all defects. Applying the cooling enhances the comprehensibility of the phase image (Figure 11b). Figure 12 illustrates the peak-to-peak amplitude. Improvements in defect detection in cooling mode (Figure 12b) are also evident in this parameter, in contrast to the mode without cooling (Figure 12a).

### 4.2. Experiment with an Excitation Period of T = 100 s

**Figure 13:**
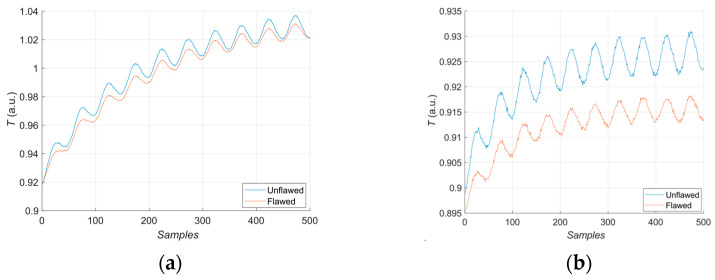
Thermal response for the flawed: (**a**) uncooled sample; (**b**) cooled sample. Excitation Period of *T* = 100 s.

**Figure 14:**
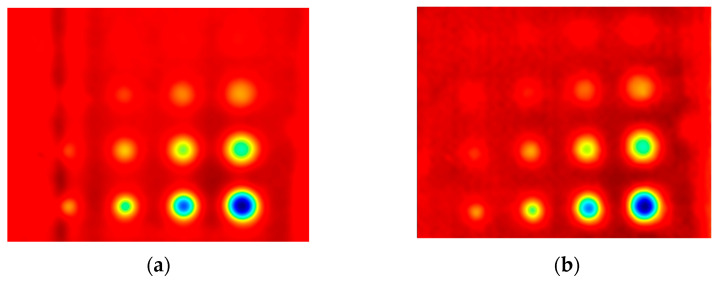
The real part of the signal: (**a**) uncooled sample; (**b**) cooled sample. Excitation Period of *T* = 100 s.

**Figure 15:**
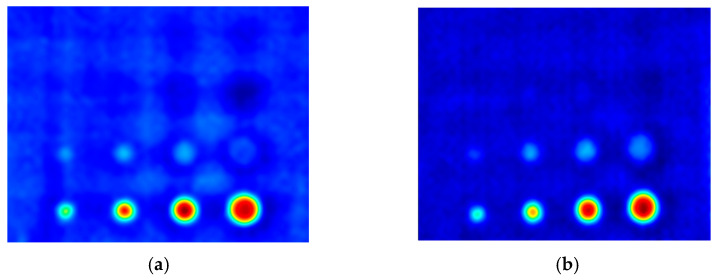
The imaginary part of the signal: (**a**) uncooled sample; (**b**) cooled sample. Excitation Period of *T* = 100 s.

**Figure 16:**
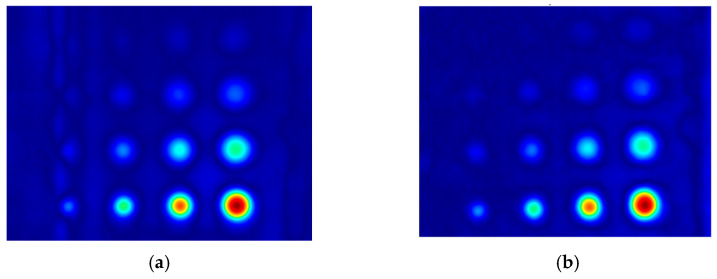
The mean amplitude of the signal: (**a**) uncooled sample; (**b**) cooled sample. Excitation Period of *T* = 100 s.

**Figure 17:**
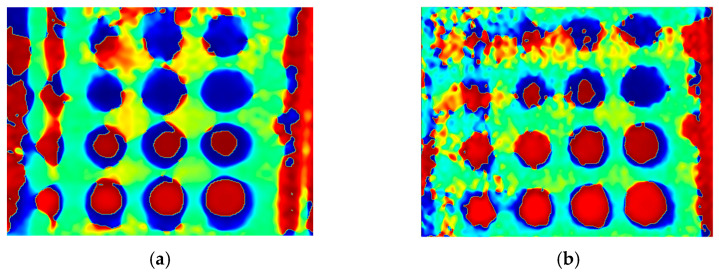
The mean phase of the signal: (**a**) uncooled sample; (**b**) cooled sample. Excitation Period of *T* = 100 s.

**Figure 18:**
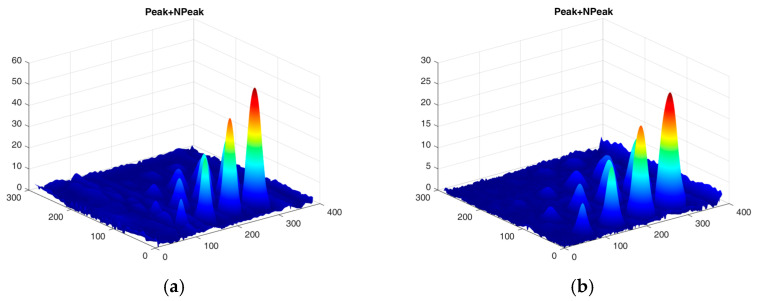
The peak-to-peak amplitude of the signal: (**a**) uncooled sample; (**b**) cooled sample. Excitation Period of *T* = 100 s. The colors correspond to the signal’s amplitude value.

The second experiment used an excitation period of *T =* 100 s. Figure 13 illustrates exemplary thermal responses for the flawed and unflawed areas of the examined sample. Similarly to the first experiment with an excitation period of 40 s, the average temperatures of the simultaneously heated and cooled sample (Figure 13b) increase at a slower rate than those of the uncooled sample (Figure 13a). Figure 14 shows the real part of the lock-in signal. As can be seen from the images, extending the period of the lock-in excitation signal from 40 to 100 s improves the detection of defects in both uncooled (Figure 14a) and parallel heating and cooling modes (Figure 14b). A similar conclusion can be drawn from Figure 15a,b, which illustrate the imaginary part of the lock-in signal. Figure 16 comprises the amplitude of the lock-in signal. Compared to the images for a period equal to 40 s, only a slight improvement in readability is apparent here. Analogously, introducing cooling parallel to the heating process (Figure 16b) instead of solely heating the sample (Figure 16a) allows for the better detectability of the flaws. Figure 17 shows the phase changes for the sample in heating mode (Figure 17a) and in heating mode with simultaneous cooling (Figure 17b). Compared with the results obtained for a period equal to 40 s, the readability of the images via the simultaneous heating–cooling improved significantly. However, the result for the heating mode remained partially blurry, so 4 of the 16 flaws are still undetectable. Figure 18a,b illustrate the peak-to-peak amplitude for both measuring modes. For this parameter, as the period of the excitation signal increased, the detection of defects improved.

## 5. Discussion

### Quantitative Analysis of Heat Excitation Modes and Excitation Periods

This study used lock-in thermography to compare two different heat excitation modes of composite materials: heating and heating with simultaneous cooling. The impact of the heatwave’s excitation period on the flaw detectability was also examined. In order to provide a quantitative comparison of excitation modes and periods, the signal-to-noise ratios for the individual flaws of the sample were calculated using the Formula (1)

(1) $$SNR=\frac{\left|\mu_{S}-\mu_{B}\right|}{\sigma_{B}}$$

where *μ_S_* is the average temperature value over the defect region; *μ_B_* is the mean temperature value over the sound region; and *σ_B_* is the standard deviation temperature value over the defect region.

**Figure 19:**
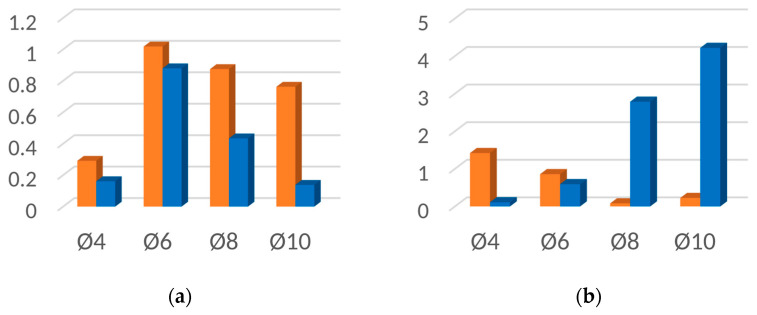
SNR for the uncooled (orange bars) and cooled (blue bars) modes for the flaw depth *d* = 1 mm and the excitation period: (**a**) *T* = 40 s; (**b**) *T* = 100 s.

**Figure 20:**
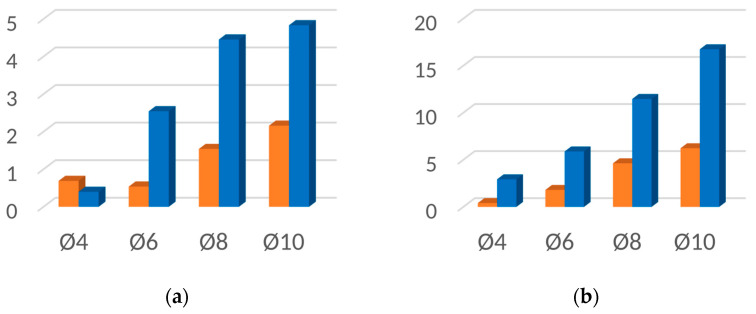
SNR for the uncooled (orange bars) and cooled (blue bars) modes for the flaw depth *d* = 1.5 mm and the excitation period: (**a**) *T* = 40 s; (**b**) *T* = 100 s.

**Figure 21:**
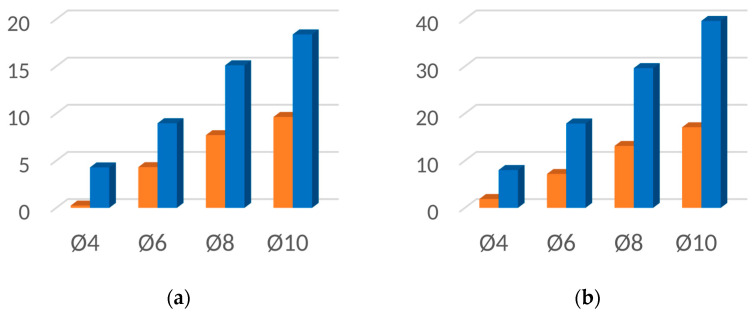
SNR for the uncooled (orange bars) and cooled (blue bars) modes for the flaw depth *d* = 2 mm and the excitation period: (**a**) *T* = 40 s; (**b**) *T* = 100 s.

**Figure 22:**
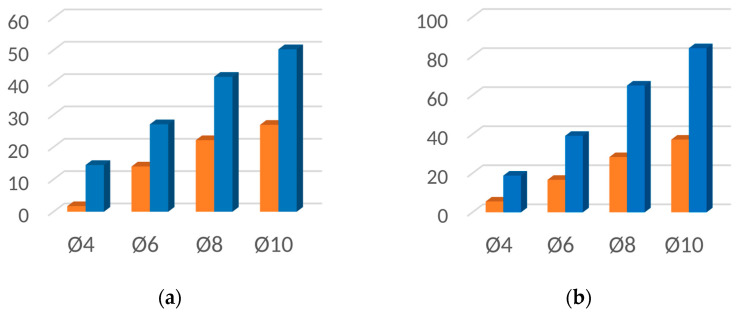
SNR for the uncooled (orange bars) and cooled (blue bars) modes for the flaw depth *d* = 3 mm and the excitation period: (**a**) *T* = 40 s; (**b**) *T* = 100 s.

Figure 19, Figure 20, Figure 21 and Figure 22 contain bar graphs of the SNR values for the heating and heating with simultaneous cooling modes. Each figure for a defined defect depth consists of two bar graphs, one for the heat excitation period of *T =* 40 s and one for *T =* 100 s.

Figure 19 illustrates the SNR for the defect depth *d* = 1 mm. For *T* = 40 s, the heating–cooling mode caused a deterioration in SNR values (Figure 19a). Extending the excitation period *T* to 100 s improved the SNR for the defect with Ø4 mm. In the heating–cooling mode, for the defects Ø4 mm and Ø6 mm, the SNR dropped, but for Ø8 mm and Ø10 mm, it improved significantly (Figure 19b). Figure 19 contains bar graphs of the SNR for the defect depth *d* = 1,5. The comparison of SNR values for the heating and heating–cooling modes for *T* = 40 s suggests that, apart from in the case of the defect Ø4, the signal over the defective area was enhanced markedly (Figure 20a). Further signal enhancement occurs for all defect radii in the case of the heating–cooling mode for *T* = 100 s (Figure 20b). As seen in Figure 21a,b, the SNR values in the case of the defect depth *d* = 2 mm can be slightly increased by extending the excitation period to 100 s. However, a considerable improvement in signal strength is obtained when applying the heating–cooling mode. Similar conclusions can be formulated for *d* = 3 mm (Figure 22a,b).

## 6. Conclusions

Lock-in thermography is a widely utilized technique for the nondestructive testing of composite structures. In the abovementioned studies, a system of lock-in thermography was built, and two modes of sample inspection were considered: heating and simultaneous heating–cooling. Particular attention was paid to studying the effect of the period of the excitation signal on detecting defects. Based on the research performed, the following conclusions can be made:

- The experimental results show that the combination of heating–cooling mode and an extended excitation period improves the SNR for most defects. However, the SNR drops for the deeper defects while introducing the heating–cooling mode. For this reason, the parameters of the proposed lock-in thermography system must be optimized to maintain high SNR for deeper defects.
- On the one hand, extending the excitation period increases the measurement time and causes the sample’s average temperature to heat up more. On the other hand, the SNR of minor defects is significantly enhanced. However, the risk of overheating the sample can be overcome by attaching a cooling unit.
- Simultaneous heating and cooling improve the detection of defects even if the excitation period is small. The cooling process reduces the sample heating rate with a more extended excitation period and makes a more significant number of defects detectable and identifiable.
- The experiment was limited to examining hidden defects in the material. In future research, it would be necessary to test the method on natural defects of shapes other than circular. Different defects, such as delamination, porosity, or void, should also be considered in future studies.
- The proposed measurement system yielded satisfactory results in diagnosing defects in glass fiber-reinforced composites. In the future research, other composite materials, such as carbon fiber-reinforced polymers, should be considered.

## Figures and Tables

**Table 1:** Selected parameters of the infrared camera.

Parameter	Value
Camera resolution	384 × 288
Frame rate	30 fps
Thermal sensitivity	≤50 mK at F/1
Spectral range	8–14 μm
Operating temperature	−10 °C~+65 °C
Scene range temperature	−10 °C~+150 °C

## Data Availability

Available on request.
